# Impact of currently prescribed lipid-lowering drugs on plasma PCSK9 concentration: single or in combination study in rats

**DOI:** 10.1186/1476-511X-13-35

**Published:** 2014-02-18

**Authors:** Yan Zhang, Jun Liu, Sha Li, Rui-Xia Xu, Jing Sun, Jian-Jun Li

**Affiliations:** 1 Division of Dyslipidemia, State Key Laboratory of Cardiovascular Disease, Fu Wai Hospital, National Center for Cardiovascular Diseases, Chinese Academy of Medical Sciences, Peking Union Medical College, BeiLiShi Road 167, Beijing 100037, China

**Keywords:** PCSK9, Statin, Lipid profile, Rat

## Abstract

**Background:**

An emerging data suggested a significant impact of statins on PCSK9 concentration, while the rapid impacts of other lipid-lowering drugs such as ezetimibe and xuezhikang alone or in combination on PCSK9 and lipid profile have not been assessed. This study aims to investigate whether an enhanced PCSK9 concentration by single or combined therapy of lipid-lowering drugs currently used precedes the changes of lipid profile in rats.

**Methods:**

Sixty-three rats were randomly divided into six groups and orally administrated with placebo (N = 13), ezetimibe 10 mg/kg daily, Xuezhikang 1200 mg/kg daily, ezetimibe 10 mg/kg plus Xuezhikang 1200 mg/kg daily, pitavastatin 10 mg/kg daily or pitavastatin 10 mg/kg plus ezetimibe 10 mg/kg daily for 3 days (N = 10 for each group respectively). Blood samples were obtained at day 3 after orally administration. Plasma PCSK9 levels were determined by ELISA and lipid profile were measured by enzymatic assay.

**Results:**

Ezetimibe, Xuezhikang and pitavastatin alone and Xuezhikang plus ezetimibe as well as pitavastatin plus ezetimibe increased PCSK9 levels by 124%, 56%, 111%, 63% and 204% respectively (p < 0.05 compared with placebo). However, Xuezhikang plus ezetimibe did not enhance greater PCSK9 levels compared to monotherapy. Ezetimibe and pitavastatin in combination induced higher PCSK9 levels than pitavastatin monotherapy or co-therapy with ezetimibe plus Xuezhikang. There was no significant difference between any groups with regard to lipid profile levels at day 3 (P > 0.05).

**Conclusions:**

Elevated PCSK9 concentration by ezetimibe, Xuezhikang and pitavastatin alone or in combination was found prior to the alterations of lipid profile in rats. Combination of Xuezhikang and ezetimibe significantly attenuated increase in PCSK9 compared to ezetimibe plus pitavastatin, suggesting that the former combination may be better than the latter in future clinical application.

## Introduction

Proprotein convertase subtilisin/kexin type 9 (PCSK9) is a secreted protease synthesized mainly in the liver, which binds directly to the epidermal growth factor repeat A of the low-density lipoprotein receptor (LDLR) and subsequently targets it for degradation [1]. This process results in increased serum LDL-cholesterol (LDL-C) levels in the circulation [2]. Gain-of-function mutations in the PCSK9 gene are associated with autosomal dominant hypercholesterolemia and premature cardiovascular disease [3,4]. Conversely, loss-of-function mutations of the PCSK9 gene linked to low LDL-C levels and a dramatically reduced risk for coronary heart disease [5]. Therefore, PCSK9 has been considered as another target for dyslipidemia and atherosclerosis [6].

Recently, several studies indicated that statins (HMG-CoA reductase inhibitors) could upregulate PCSK9 levels in humans [7-9], which may attenuate the beneficial effects of statins [10]. It was recently postulated that statins increase the activity of sterol regulator element–binding protein 2 (SREBP-2), which is a transcription factor that activates both the LDLR and PCSK9 genes [11], and eventually induces elevated expression and secretion of PCSK9 protein. In addition, other common prescribed lipid-lowering drugs, ezetimibe, Xuezhikang are widely used as alone or in combination for the management of dyslipidemia. For example, ezetimibe is an inhibitor of intestinal cholesterol absorption, which could decrease LDL-C by approximately 20% when administered alone [12] and when combined with statin could achieve 15% more decrease in LDL-C concentrations compared with statin alone [13]. However, a few reports about whether ezetimibe could exert similar effects on PCSK9 as statins are inconsistent [14,15].

Extracts of red yeast rice have been widely used for therapy of patients with cardiovascular disorders in China for centuries. Xuezhikang, a partially purified extract of fermented red yeast rice, contains 13 kinds of natural statins (high amounts of lovastatin), unsaturated fatty acids, ergosterol, flavonoids, and some other components [16,17]. A systematic review demonstrated that Xuezhikang exerted significant lipid-lowering effects in the treatment of hyperlipidemia [18]. Recently, clinical studies indicated that Xuezhikang could be used as an alternative therapy for patients intolerant of statin [19]. However, there are limited data to evaluate the impact of Xuezhikang on the PCSK9 levels. Although previous studies have implied that lipid-lowering drugs have short-term effects on PCSK9 in humans [20], study investigating whether the impact of currently used lipid-lowering drugs on PCSK9 levels prior to the changes of lipid profile is still limited. Moreover, the impacts of lipid-lowering drugs in combination on PCSK9 has less been evaluated till now.

Therefore, the aim of the present study was to evaluate: 1) whether the increase of PCSK9 concentration is prior to the decrease of LDL-C levels; 2) the rapid effects of different lipid-lowering drugs on PCSK9 concentration; 3) the different impacts of lipid-lowering drugs in combination on PCSK9 concentration.

## Materials and methods

### Animal preparation

Male Sprague–Dawley rats, weighing 180–220 g, were fed in a temperature-conditioned (22-24°C) room with alternating 12 h light/dark cycles and allowed them free access to food and water for 3 days to get familiar with the environment. At the start of the study, 63 rats were randomly assigned to six groups: (1) placebo group (N = 13, orally administrated with normal saline), (2) ezetimibe group (N = 10, orally administrated with ezetimibe 10 mg/kg daily), (3) Xuezhikang group (N = 10, orally administrated with Xuezhikang 1200 mg/kg daily), (4) ezetimibe plus Xuezhikang group (N = 10, orally administrated with ezetimibe 10 mg/kg plus Xuezhikang 1200 mg/kg daily), (5) pitavastatin group (N = 10, orally administrated with pitavastatin 10 mg/kg daily), and (6) pitavastatin plus ezetimibe group (N = 10, orally administrated with pitavastatin 10 mg/kg plus ezetimibe 10 mg/kg daily). This study was conducted in accordance with the Guide for the Care and Use of Laboratory Animals published by the National Institutes of Health, and all experimental procedures and protocols were approved by the Care of Experimental Animals Committee of Fuwai Hospital, Chinese Academy of Medical Sciences and Peking Union Medical College.

### Blood sample and analysis

After 3 days orally administration, prior to sacrifice, 2 ml fasting blood were collected from the inferior vena cava and transferred to K2 EDTA tubes. Immediately after blood sampling, the animals were euthanized by an overdose of pentobarbital sodium. The blood samples were centrifuged, and the plasma was stored at −80°C until the analyses were performed. Concentrations of serum total cholesterol (TC), triglycerides (TG), high density lipoprotein cholesterol (HDL-C) and LDL-C were determined on an automatic biochemistry analyzer (Hitachi 7150, Tokyo, Japan). Plasma PCSK9 concentrations were measured using a high-sensitivity, quantitative sandwich enzyme immunoassay (Quantikine ELISA, R&D Systems Europe Ltd). The lower limit of detection was 0.096 ng/ml.

### Statistical analysis

Continuous variables were expressed as mean ± standard deviation (SD) and categorical variables were presented as frequent count and percentages. The comparison of inter-group analysis of PCSK9 and lipid profile levels was performed by unpaired two-tailed t-test and one way ANOVA. A P value of less than 0.05 was considered statistical significance. The SPSS 19.0 statistical software package (SPSS Inc., Chicago, IL, USA) was used for all of the statistical analysis.

## Results

### The changes of lipid profile

As shown in Table 1, there was no significant difference in lipid profile among rats receiving placebo, ezetimibe, Xuezhikang, pitavastatin, ezetimibe plus Xuezhikang, or pitavastatin plus ezetimibe at day 3 (p > 0.05).

**Table 1:** Laboratory data

**Parameters**	**Placebo (N = 13)**	**Ezetimibe (N = 10)**	**Xuezhikang (N = 10)**	**Xuezhikang + ezetimibe (N = 10)**	**Pitavstatin (N = 10)**	**Pitavstatin + ezetimibe (N = 10)**
PCSK9 ng/ml	296.29 ± 112.95	664.76 ± 331.73*	461.20 ± 124.96*	482.86 ± 130.29*	626.16 ± 221.48*	901.35 ± 235.56*^#Δ^
TG mmol/l	0.76 ± 0.72	0.51 ± 0.17	1.38 ± 0.12	1.26 ± 0.29	0.44 ± 0.12	0.91 ± 0.18
TC mmol/l	2.13 ± 0.26	2.44 ± 0.40	2.25 ± 0.37	2.19 ± 0.40	2.50 ± 0.40	2.39 ± 0.33
HDL-C mmol/l	1.04 ± 0.29	1.31 ± 0.29	1.04 ± 0.29	0.94 ± 0.20	1.25 ± 0.27	1.22 ± 0.27
LDL-C mmol/l	0.53 ± 0.14	0.51 ± 0.15	0.45 ± 0.07	0.42 ± 0.04	0.64 ± 0.18	0.48 ± 0.09

Data are expressed as means ± SD. * p < 0.05 versus placebo group. ^#^ p < 0.05 versus pitavastatin group. ^Δ^p < 0.05 versus Xuezhikang + ezetimibe group. PCSK9 = proprotein convertase subtilisin/kexin type 9; TG = triglycerides; TC = total cholesterol; HDL-C = high density lipoprotein cholesterol; LDL-C = low density lipoprotein cholesterol.

### Rapid effects of lipid-lowering drugs on plasma PCSK9 concentrations

**Figure 1:**
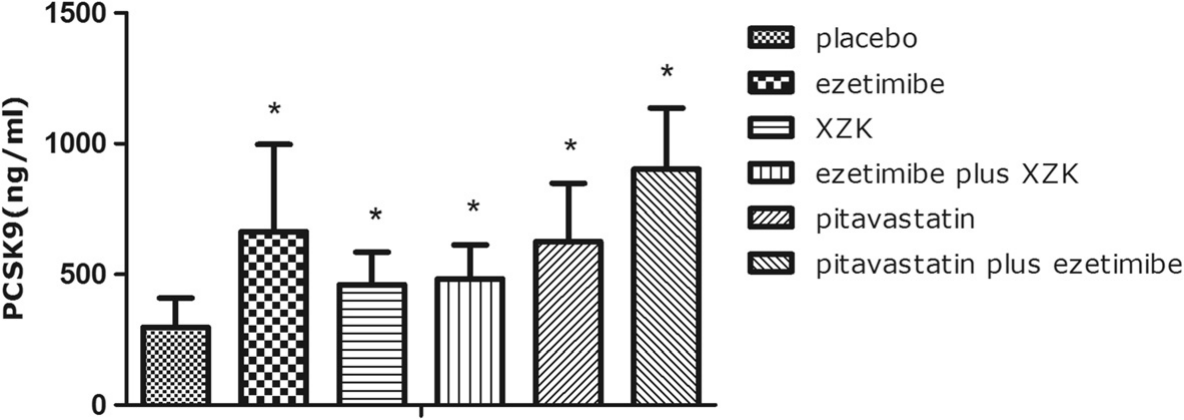
**PCSK9 levels during the treatment with placebo, ezetimibe, Xuezhikang, Xuezhikang plus ezetimibe, pitavastatin and pitavastatin plus ezetimibe.** * p < 0.05 versus placebo group. XZK: Xuezhikang.

**Figure 2:**
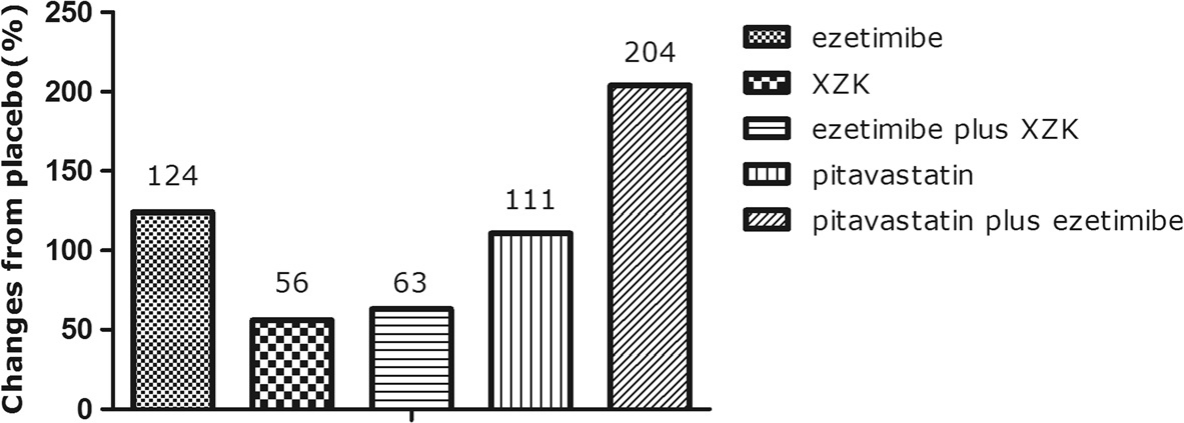
**Mean percentage changes from placebo in PCSK9 levels during the treatment with ezetimibe, Xuezhikang, Xuezhikang plus ezetimibe, pitavastatin and pitavastatin plus ezetimibe.** XZK: Xuezhikang.

As shown in Figures 1 and 2, at day 3, PCSK9 levels increased from 296.29 ± 112.95 ng/ml to 664.76 ± 331.73 ng/ml in rats receiving ezetimibe 10 mg/kg daily treatment (124%; p < 0.05 vs. placebo). In rats treated with Xuezhikang 1200 mg/kg daily, PCSK9 levels increased from 296.29 ± 112.95 ng/ml to 461.20 ± 124.96 ng/ml (56% increase, p < 0.05 vs. placebo). In rats with pitavastatin 10 mg/kg daily treatment, PCSK9 increased from 296.29 ± 112.95 ng/ml to 626.16 ± 221.48 ng/ml (111% increase, p < 0.05 vs. placebo). The combination of ezetimibe 10 mg/kg daily plus Xuezhikang 1200 mg/kg daily resulted in an increase of PCSK9 levels by 63% (296.29 ± 112.95 to 482.86 ± 130.29 ng/ml). Pitavastatin 10 mg/kg daily and ezetimibe 10 mg/kg daily in combination resulted in an increase of PCSK9 by 204% (296.29 ± 112.95 to 901.35 ± 235.56 ng/ml; p < 0.05 vs. placebo).

### Combination of ezetimibe plus Xuezhikang, ezetimibe plus pitavastatin on plasma PCSK9 concentrations

**Figure 3:**
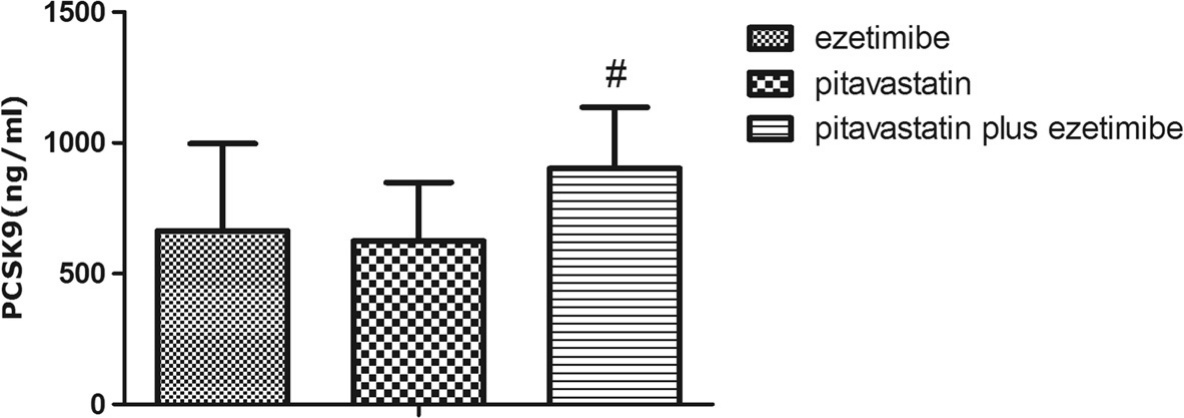
**PCSK9 levels during the treatment with ezetimibe, pitavastatin and pitavastatin plus ezetimibe. **^#^ p < 0.05 versus pitavastatin group. XZK: Xuezhikang.

**Figure 4:**
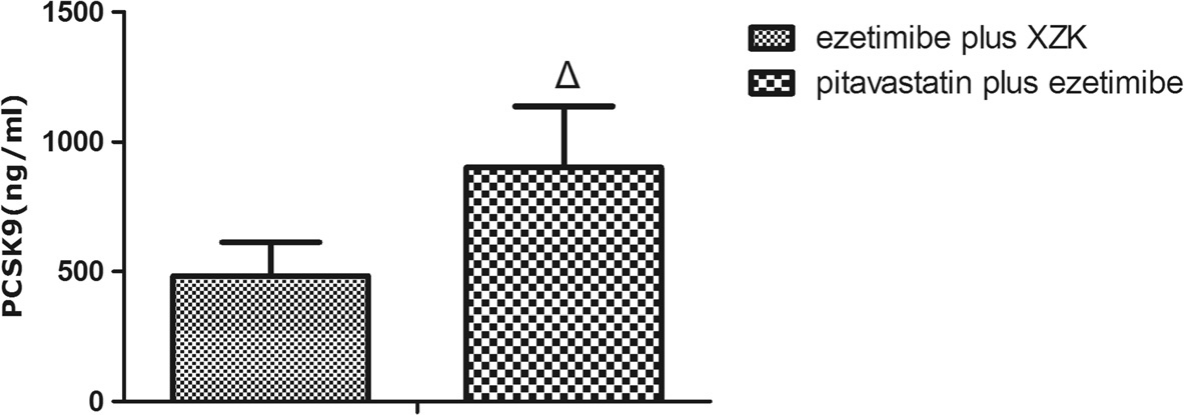
**PCSK9 levels during the treatment with Xuezhikang plus ezetimibe and pitavastatin plus ezetimibe. **^Δ^p < 0.05 versus Xuezhikang plus ezetimibe group. XZK: Xuezhikang.

As shown in Table 1, at day 3, in combination of ezetimibe and Xuezhikang did not induce greater increase of PCSK9 concentrations than monotherapy with either agent (p > 0.05). However, as shown in Figures 3 and 4, the PCSK9 concentrations among rats treated with ezetimibe plus pitavastatin was much higher than monotherapy with pitavastatin or the co-therapy of ezetimibe plus Xuezhikang (p < 0.05).

## Discussion

The current experiment explored the rapid effects of the 3 currently prescribed lipid-lowering drugs, pitavastatin, ezetimibe and Xuezhikang, alone and in combination, on PCSK9 and lipid profile concentrations in rats in a very short period. The main finding of this study is that ezetimibe, Xuezhikang and pitavastatin alone and Xuezhikang plus ezetimibe as well as pitavastatin plus ezetimibe, all could significantly increase the PCSK9 levels at day 3, when the lipid profile had no obvious decreases. In addition, combination of ezetimibe 10 mg/kg and Xuezhikang 1200 mg/kg daily did not further enhance PCSK9 levels compared to the monotherapy, while ezetimibe 10 mg/kg and pitavastatin 10 mg/kg daily in combination induced higher PCSK9 levels than monotherapy with pitavastatin or co-therapy with ezetimibe plus Xuezhikang.

It has been well established that PCSK9 promotes the degradation of LDLR and could limit the beneficial effects of lipid-lowering drugs [1]. Several studies indicated that lipid-lowering drugs could elevate plasma PCSK9 concentrations and may have rapid impact on PCSK9 levels [20]. In our present study, we confirmed previous studies and provided novel additional important information regarding the lipid-lowering drugs on plasma PCSK9. This study, for the first time, indicated that the enhancement of PCSK9 levels preceded the changes of lipid profile. Therefore, it is reasonable to deduce that patients with dyslipidemia may get more benefits from PCSK9 inhibitors, such as monoclonal antibody [21], if it be used before the application of lipid-lowering drugs in clinical practice. Although commonly prescribed lipid-lowering drugs may up-regulate both the LDLR and PCSK9 genes by activating SREBP-2 [11], the PCSK9 gene might respond much earlier than the LDLR gene, that may partly of the reason why the changes of PCSK9 levels preceded the lipid profile.

Currently, combination of two different lipid-lowering drugs is frequently prescribed in controlling dyslipidemia. Statin plus ezemtibe in combination is the most commonly used style. In recent years, several researchers have described the combination of statin and ezetimibe on the increase of PCSK9 levels in humans. A recent study conducted by Davignon et al. [22] demonstrated that patients treated with statins alone had a 45% increase in PCSK9 levels and those treated with statin plus ezetimibe showed an approximately 77% increase in PCSK9 concentrations. In our study, the PCSK9 levels increased by 111% in rats administrated with pitavastatin 10 mg/kg daily, while added ezetimibe 10 mg/kg daily to pitavastatin, the PCSK9 levels enhanced by 204%. However, little was known about the reason why ezetimibe causes a further increase in PCSK9 levels. Since ezetimibe exerts its effect by binding to Niemann-Pick C1-Like 1, and thereby inhibiting intestinal cholesterol absorption and subsequently results in reduced hepatic cholesterol [23], it may be possible that this process is accompanied by a feedback mechanism on transcription factor SREBP-2. Furthermore, in mice models, Brandon Ason et al. [15] applied qRT-PCR to analyze the expression of 361 genes involved in hepatic lipid metabolism and found that many genes within the SREBP-2 pathway were induced following ezetimibe treatment (2.5-fold average induction relative to control) and were even further induced by ezetimibe plus rosuvastatin combination treatment (11-fold average induction relative to control). This study provides theoretical evidences about the impact of ezetimibe on plasma PCSK9 levels. In contrast, Heiner K. Berthold et al. [14] indicated that when added to simvastatin, ezetimibe does not cause an incremental increase in PCSK9 concentrations, which conflicted with our observations. However, this study explored the 14 days effect of ezetimibe-statin in combination on plasma PCSK9 levels in humans, and different kinds of statins were used in the two studies. Even so, for the sake of exploring a thorough explanation of the mechanism, further studies are warranted.

In our study, we also observed that combination of ezetimibe 10 mg/kg and Xuezhikang 1200 mg/kg daily did not bring a greater increase in PCSK9 levels than monotherapy, and the PCSK9 levels was significantly lower than co-therapy with ezetimibe plus pitavastatin. Therefore, for patients who need an intensive lipid-lowering therapy, the combination of Xuezhikang and ezetimibe would be a better alternative therapy. This combination could be a better strategy not only for exerting good lipid-lowering effect on LDL-C, but also for mitigating the adverse effects of lipid-lowering drugs on PCSK9 in controlling dyslipidemia and atherosclerosis. At the present time, with regarding to the mechanism by which ezetimibe plus Xuezhikang causes an attenuated increase in PCSK9 levels compared to monotherapy and co-therapy with ezetimibe plus pitavastatin is unclear, but it may likely associated with the chemical constituents of Xuezhikang. Xuezhikang, a Chinese traditional medicine, is widely used as a lipid-lowering drug, and its main components contain lovastatin, as well as other useful substances [16-18]. Recent studies including our data have shown that Xuezhikang has many pleiotropic effects, which could effectively modify not only lipid profile but also inflammatory markers [24]. The interactions among different constituents of Xuezhikang and ezetimibe may influence the effect on PCSK9 levels, but clinical trials and molecular mechanisms are needed in the further studies.

For the past decade, PCSK9 has gained tremendous attention. It is well-established that statin treatment elevates plasma PCSK9 levels and, on the contrary, inhibition of PCSK9 function enhances the lipid-lowering effects of statins [25,26]. Among the various PCSK9 inhibitors, human data are available for monoclonal antibodies against PCSK9 of which the two most advanced are SAR236553/REGN727 and AMG 145. In a 12-week phase II study of patients with LDL-C levels ≥ 100 mg/dl on a stable dose of atorvastatin (10, 20, or 40 mg/day), add-on of SAR236553/REGN727 (doses 50–150 mg) administered subcutaneously every 2 weeks resulted in LDL-C reductions of 40–72% [27]. The LAPLACE-TIMI trial evaluated patients with documented hypercholesterolemia, while on a statin with or without ezetimibe treatment. The data had shown reductions of LDL-C by 41.8% to 66.1% when administrated AMG 145 at 70, 105 and 140 mg every 2 weeks, with acceptable safety and tolerability [21]. Combining our findings and previous studies together may suggest that, not only statins, but also ezetimibe and Xuezhikang could increase plasma PCSK9 levels, and this effects occurred before the changes of lipid-profile, which strongly suggest that PCSK9 inhibitors should be combined with the currently prescribed lipid-lowering drugs to result in further LDL-C lowering effect, and it would be better to be used prior to lipid-lowering drugs. Moreover, our data also provides additional information with respect to combination of ezetimibe plus Xuezhikang may be a better choice than ezetimibe plus pitavastatin in clinical practice.

There are several limitations in the present study. First, we did not observe the long-term effect of different lipid-lowering drugs, alone and in combination, on plasma PCSK9 and lipid-profile concentrations. Second, we did not explore the molecular mechanisms of the phenomenon. Hence, further studies are needed.

## Competing interests

The authors declare that they have no competing interests.

## Authors’ contributions

ZY and LJ completed the project, and analyzed the data, and wrote the manuscript. LJ-J established the study, interpreted the data, and contributed to reviewing/editing the manuscript. LS, XR-X, and SJ contributed to assay and analyzing the data. All authors read and approved the final manuscript.
